# Ligustrazinyl amides: a novel class of ligustrazine-phenolic acid derivatives with neuroprotective effects

**DOI:** 10.1186/s13065-015-0084-5

**Published:** 2015-03-04

**Authors:** Guoliang Li, Xin Xu, Kuo Xu, Fuhao Chu, Jixiang Song, Shen Zhou, Bing Xu, Yan Gong, Huazheng Zhang, Yuzhong Zhang, Penglong Wang, Haimin Lei

**Affiliations:** School of Chinese Pharmacy, Beijing University of Chinese Medicine, No.6 Wangjing Middle Ring South Road, Beijing, Chaoyang District China; Department of Pathology, Beijing University of Chinese Medicine, No.11 North Third Ring Road, Beijing, Chaoyang District China

**Keywords:** Ligustrazine derivative, Phenolic acid, Neuroprotective effect, PC12 cell

## Abstract

**Background:**

Ligustrazine has potent effects of thrombolysis, neuroprotection and vascular protection, which were important for effectively protecting the nervous system. Previous study in our laboratory reported that ligustrazine-benzoic acid derivatives have been shown to exhibit beneficial effect against CoCl_2_-induced neurotoxicity in differentiated PC12 cells. To further improve ligustrazine’s neuroprotection, we integrated the ligustrazine and phenolic acid fragments into one molecule via an amide bond based on structural combination.

**Results:**

In this study, 12 novel ligustrazine-phenolic acid derivatives were synthesized and nine others were prepared by improved methods. Furthermore, these compounds were evaluated for their protective effects against CoCl_2_-induced neurotoxicity in differentiated PC12 cells. The amides conjunctional derivatives exhibited promising neuroprotective activities in comparison with ligustrazine. In addition, the most active congener (E)-3-(2,3,4-trimethoxyphenyl)-N-((3,5,6-trimethylpyrazin-2-yl)methyl)acrylamide (**L10**, EC_50_ = 25 μM), which is 2 times higher than that of ligustrazine, may be a potential candidate for intervention in neurological diseases. Structure-activity relationship was discussed briefly.

**Conclusions:**

Results of series of ligustrazinyl amides enrich the study of ligustrazine derivatives with neuroprotective effects. Our completed work supports that the attempt to apply structure combination to discover more efficient neuroprotection lead compounds is viable.

**Graphical Abstract:**
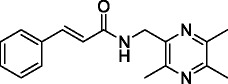
Ligustrazinyl Amides **L1-L21** with Neuroprotective Effects.

## Background

Neurological disorders, such as Stroke, Alzheimer’s disease (AD) and Parkinson’s disease (PD), threaten millions of patients with ever growing numbers in ageing societies [1-3]. To discover drugs with nerve functional recovery as a treatment or prevention of neurological disorder is of great significance [4]. To date, despite the remarkable progress achieved in theory, effective approaches to recover damaged nerve are not yet to be found [1,5]. Therefore, to discover more effective drugs for the treatment in injured nerve cell remains an important area of drug discovery [6,7].

Nowadays, many new drugs have been generated from natural products [8-11]. Ligustrazine (2,3,5,6-tetramethylpyrazine, TMP), derived from the traditional Chinese medicine Rhizoma Chuanxiong (*Ligusticum chuanxiong* Hort.) which was widely used to treat Stroke and cerebrovascular disease (CVD) in China [12,13]. Recent studies have indicated that ligustrazine has potent effects of thrombolysis, neuroprotection and vascular protection, which were important for effectively protecting the nervous system [14-21]. In addition, many phenolic acid ingredients, such as caffeic acid, protocatechuic acid, salicylic acid, ferulic acid, vanillic acid, etc., also showed interesting neuroprotective activities [21-24].

To further improve ligustrazine’s neuroprotective effect, inspired by the potent neuroprotective effects of ligustrazine-benzoic acid derivatives, we integrated the ligustrazine and phenolic acid fragments into one molecule based on structural combination [17,21,25]; a series of novel ligustrazine-benzoic analogues was constructed via an amide bond rather than an ester bond in our previous research on ligustrazine-benzoic acid derivatives. A recent study has reported that part of ligustrazinyl amides congener structures exhibited good proliferative activities on human umbilical vascular endothelial cells (HUVECs) [25]. Their protective effects against neurotoxicity were evaluated in differentiated PC12 cells. Structure-activity relationship was discussed briefly.

## Results and discussion

### Chemistry

**Scheme 1:**
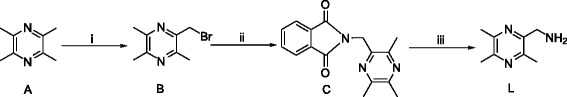
**Synthetic routes to ligustrazine intermediate L.** Reagents and Conditions: (i) CCl_4_, NBS, hv, reflux, 2 h, 65%; (ii) CH_3_CN, phthalimide potassium, reflux, 2 h, 64%; (iii) CH_3_CH_2_OH, N_2_H_4_ · H_2_O, reflux, 5 h, 88%.

**Scheme 2:**
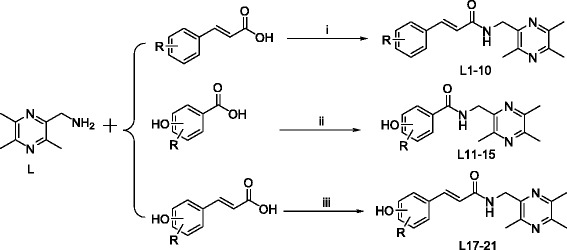
**Synthetic routes to ligustrazine derivatives L1–L15, L17–L21.** Reagents and Conditions: (i) anhydrous CH_2_Cl_2_, EDCI/(CH_3_CH_2_)_3_ N, r.t., 12 h; (ii) anhydrous DMF (L11 anhydrous CH_2_Cl_2_), EDCI/HOBt, r.t., 12 h; (iii) anhydrous DMF (L21 DMI), EDCI/HOBt, r.t., 12 h.

**Scheme 3:**
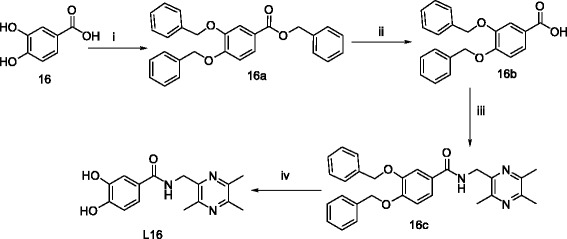
**Synthetic routes to ligustrazine derivatives L16.** Reagents and Conditions: (i) DMF, benzyl bromide, K_2_CO_3_, 85°C, 12 h; (ii) H_2_O/CH_3_CH_2_OH, 10% KOH, 70°C, 2 h; (iii) TMP-NH_2_, DMF, EDCI/HOBt, r.t., 12 h; (iv) CH_3_OH, Pd/C, H_2_, r.t., 12 h.

All the target compounds were synthesized via the routes outlined in Scheme 1, Scheme 2 and Scheme 3. The key intermediate (3,5,6-trimethylpyrazin-2-yl)methanamine (**L**) was prepared according to our previous study with minor improvements. Compound **B** (TMP-Br) was synthesized from anhydrous ligustrazine and N-bromosuccinimide (NBS) in carbon tetrachloride via free radical reaction, the crude product was used directly in the next reaction without further purification. The mixture of TMP-Br and phthalimide potassium in CH_3_CN that was refluxing for 2 h gave compound **C**. Intermediate **L** was obtained by reaction of **C** and 80% hydrazine hydrate in absolute ethanol refluxing for 5 h.

The single-step coupling reaction between **L** and the cinnamic acids were performed using EDCI and (CH_3_CH_2_)_3_ N in anhydrous CH_2_Cl_2_, to afford ligustrazine derivatives (**L1**–**L10**, as shown in Method 1 of Scheme 2). In Method 2 and 3, the carboxylic acids and HOBt were firstly transformed to active ester in the presence of EDCI, and then reacted with compound **L**, obtaining the target compounds (**L11**–**15**, **L17**–**21**).

In Scheme 3, the starting compound **16** was first perbenzylated and then transformed to free carboxylic acid. The coupling reaction between **L** and the hydroxyl-perbenzylated benzoic acid **16b** was performed using EDCI and HOBt in anhydrous CH_2_Cl_2_. A final deprotection step afforded the targeted compound **L16**. The chemical structures of all target compounds (Table 1) were confirmed by ^1^H-NMR, ^13^C-NMR and high resolution mass (HRMS).

**Table 1:** **The structures of ligustrazine derivatives L1–21**

**Structure**	**Yield**	**Structure**	**Yield**
**L1**	90.7%	**L2**	83.5%
**L3**	86.3%	**L4**	85.8%
**L5**	90.1%	**L6**	83.6%
**L7**	91.3%	**L8**	88.5%
**L9**	81.6%	**L10**	85.2%
**L11**	68.9%	**L12**	81.8%
**L13**	76.9%	**L14**	78.4%
**L15**	84.3%	**L16**	93.7%
**L17**	71.3%	**L18**	74.8%
**L19**	77.2%	**L20**	80.1%
**L21**	64.3%		

### Biological activities

#### Protective effect on injured neuronal-like PC12 cells

Setting ligustrazine as the positive control drug, all the synthesized compounds were tested for their protective effects on neuronal-like PC12 cells damaged by CoCl_2_. The proliferation rates (P%) of injured PC12 cells were assessed by methyl thiazolyl tetrazolium (MTT) assay. The proliferation rates (%) at different concentration and 50% effective concentrations (EC_50_) for protecting damaged PC12 cells of the ligustrazine derivatives were outlined in Table 2.

**Table 2:** **EC**_**50**_ **of the ligustrazine derivatives for protecting damaged PC12 cells**

**Compound**	**Proliferation rate (%)**					**EC**_**50**_ **(μM)**
	**60 μM**	**30 μM**	**15 μM**	**7.5 μM**	**3.75 μM**	
**L1**	−20.01	−14.71	5.47	−8.63	−15.83	150
**L2**	18.94	18.52	12.48	2.80	−10.53	74
**L3**	38.69	14.19	12.04	−0.57	−7.52	64
**L4**	8.91	9.20	11.86	4.92	1.61	77
**L5**	3.28	4.31	13.63	9.98	1.79	78
**L6**	8.68	30.75	9.73	9.06	3.07	62
**L7**	7.63	9.29	17.83	11.37	0.51	71
**L8**	3.00	7.39	54.75	35.16	0.06	46
**L9**	6.05	7.50	−3.24	−3.46	−8.38	102
**L10**	47.63	38.14	36.80	35.01	21.74	25
**L11**	46.64	43.34	33.42	24.46	22.52	27
**L12**	26.42	19.94	13.47	13.29	10.75	53
**L13**	3.35	4.89	40.02	14.72	−6.53	64
**L14**	−13.55	9.81	16.80	1.94	−11.97	98.
**L15**	−1.40	17.23	25.56	10.54	−19.67	92
**L16**	−8.69	−2.40	13.41	−1.28	−1.36	103
**L17**	11.58	28.77	39.01	10.12	−20.20	60
**L18**	0.05	0.31	8.58	−0.51	−8.65	101
**L19**	17.95	19.80	22.80	24.53	24.06	44
**L20**	13.94	19.41	25.05	28.12	34.42	40
**L21**	27.59	28.12	29.80	25.51	24.52	36
**TMP**	14.71	12.11	11.76	10.60	9.44	65

From the obtained results, it was observed that ligustrazine and most of its derivatives presented protective effects on injured differentiated PC12 cells, and several ligustrazine derivatives exhibited competitive positive activities (with lower EC_50_ values) than TMP (EC_50_ = 65 μM). Among them, **L10** and **L11** displayed promising neuroprotective activities (EC_50_ = 25, 27 μM, respectively), in which compound **L10** presented 2 times higher potency than TMP.

Among **L1**–**L10**, compounds that introduced methyl and methoxy group on the phenyl ring performed better neuroprotective activities than **L1** without any group substituted. It can be concluded that with the increase of the number of methoxy group at the phenyl moiety, the activities increase considerably (**L10** > **L3, L6**, **L8** > **L4**, **L5**, **L7**). However, this rule did not work in the case of **L9** (EC_50_ = 102 μM) on the injured PC12 cells model. The structure-activity relationship analysis was consistent with the protective effects of ligustrazine-based stilbene derivatives on damaged ECV-304 cells [26]. These findings may provide a new framework for the design of new ligustrazine derivatives as neuroprotective drugs.

It should be noticed that ligustrazinyl amides **L12**–**L16** with phenolic hydroxyl substituted approximately followed a tendency in activity 4-OH, 3-OCH_3_ > 4-OH > 2-OH > 3-OH > 3, 4-OH. This was similar to our previous research that the ligustrazine-benzoic acid derivatives were synthesized via an ester bond [21]. Moreover, compounds **L15** and **L11** were derived from salicylic acid and acetylsalicylic acid, respectively. But **L11** (EC_50_ = 27 μM) displayed observable protective action, and it is much better than **L15** (EC_50_ = 92 μM). This implied that acetyl group may be an effective group, led to improvement of the neuroprotective activities. In addition, the introduction of trans olefinic bond may contribute to enhance the neuroprotective activity, such as **L20** > **L12**, **L17** > **L13**, **L19** > **L15** and **L21** > **L16**.

Furthermore, previous studies proved that compounds **L2**, **L4**, **L19** and **L20** could stimulate the proliferation of cultured human umbilical vascular endothelial cells [25]; current study also exhibited that **L2**, **L4**, **L19** and **L20** exhibited neuroprotective activities. Based on the above evidences, we reason that the new synthetic ligustrazinylated derivatives possess multiple pharmacological activities such as neuroprotection and protection against vascular endothelial cell injury, suggesting that they may be more efficacious than either a neuroprotective agent or vascular protective drug alone.

### Effects of L10 on PC12 cells in morphology

**Figure 1:**
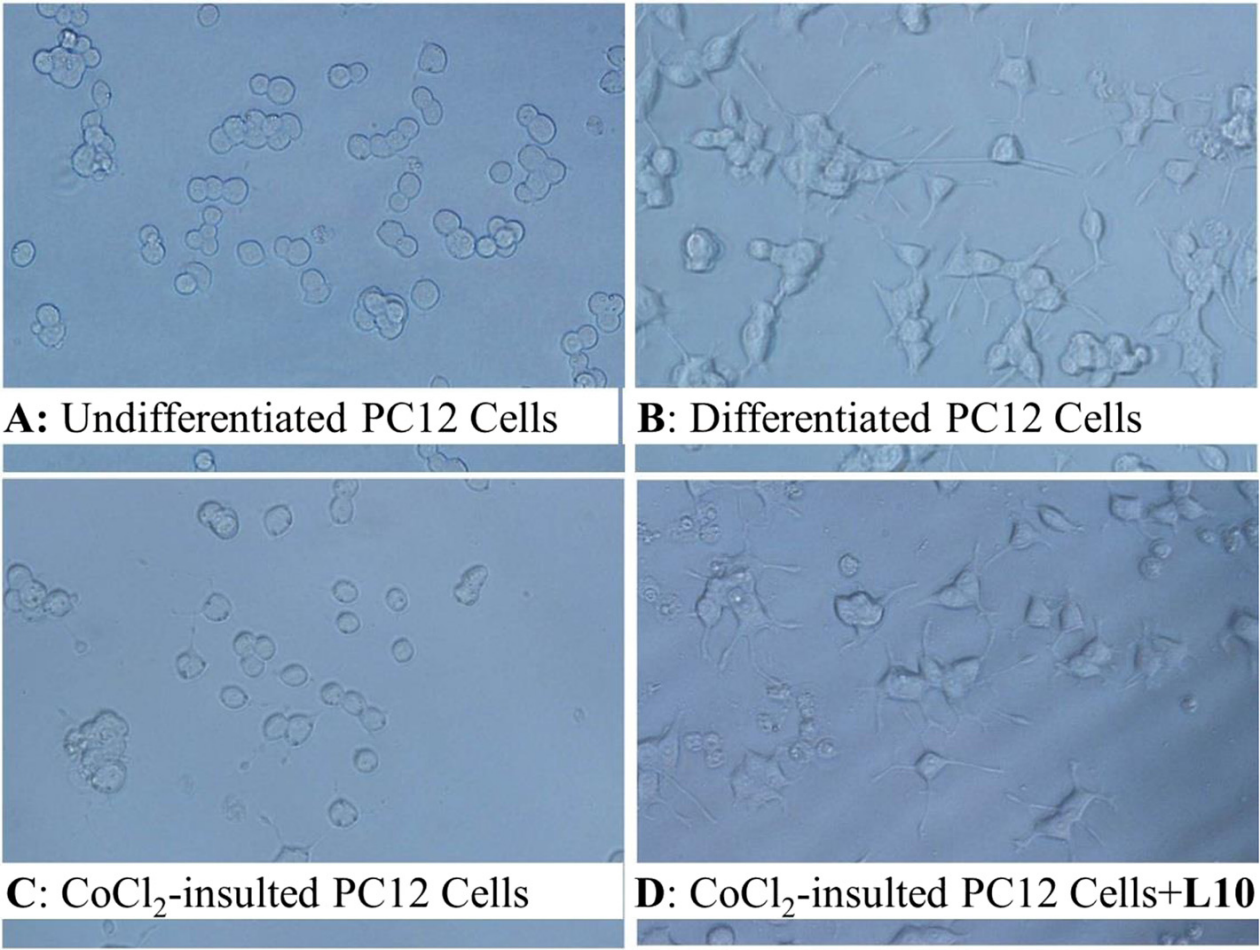
**Effects of L10 on differentiated PC12 cell injury in morphology (×200). (A)** Control PC12 cells maintained under normal conditions. **(B)** PC12 cells exposed to NGF. **(C)** Differentiated PC12 cells exposed to CoCl_2_ insult. **(D)** Differentiated PC12 cells pre-incubated with L11 then exposed to CoCl_2_ insult.

Observed under optical microscopy (OLMPUS, Japan), as shown in Figure 1-A, we found that undifferentiated PC12 cells that maintained under normal conditions were small and proliferated to form clone-like cell clusters without neural characteristics. By exposure to NGF, normal differentiated PC12 cells showed fine dendritic networks similar to those nerve cells (Figure 1-B). In contrast, CoCl_2_-insulted differentiated PC12 cells developed mild cell body swelling and some cells shrunk the dendritic networks even lost neurites demonstrating round shape (Figure 1-C). Pretreatment with **L10** alleviated morphological manifestations of cells damage compared to model cells (Figure 1-D).

## Conclusions

In this work, 21 novel ligustrazine-phenolic acid derivatives were designed, synthesized and biologically evaluated for their protective effects against CoCl_2_-induced neurotoxicity in differentiated PC12 cells. The biological results have demonstrated that most of ligustrazine derivatives exhibited better neuroprotective activities in comparison with ligustrazine. In addition, the most active congener **L10** (EC_50_ = 25 μM), which is 2 times higher than that of ligustrazine, may be a potential candidate for intervention in neurological diseases. Furthermore, structure-activity relationship was discussed briefly.

Altogether, results of series of ligustrazinyl amides enrich the study of ligustrazine derivatives with neuroprotective effects. Our completed work supports that the attempt to apply structure combination to discover more efficient neuroprotection lead compounds is viable. Furthermore, to pursue the optimized neuroprotective agents, ligustrazine-cinnamic acid ether and ligustrazine-cinnamic acid ester derivatives’ neuroprotective effects are ongoing in our lab.

## Experimental section

### Chemistry

#### Materials and methods

Reactions were monitored by TLC which was performed on silica gel GF254 (Qingdao Haiyang Chemical Co., China) and spots were visualized by modified bismuth potassium iodide or by irradiation with UV light (254 nm). Nuclear magnetic resonance spectra were recorded using a Bruker AVANCE 500 NMR spectrometer (Fällanden, Switzerland) in the indicated solvents. Chemical shifts are expressed in δ (ppm) relative to tetramethylsilane (TMS). Coupling constants are reported in Hertz (Hz). HRMS spectra were recorded on a Thermo Scientific™ LTQ Orbitrap XL hybrid FTMS instrument (Thermo Technologies, USA). Melting points (uncorrected) were metered on an X-5 micro melting point apparatus (Beijing, China). Flash column chromatography was performed on 200–300 mesh silica gel. All chemicals used were analytical. Solvents were reagent grade or high-performance liquid chromatography grade, and when necessary, were dried by standard methods. Concentration of the reaction solutions involved the use of rotary evaporator at reduced pressure. The yields were calculated by the last step reaction. Among all target compounds, **L1**, **L2**, **L4**, **L8**, **L11**, **L13**, **L15**, **L17**, **L20** were reported in Liu’s research [25]. Therefore, HRMS were supplemented to confirm the chemical structures.

### Preparation of (3,5,6-trimethylpyrazin-2-yl)methanamine (L)

The target compound **L** was synthesized by three steps. Compound **B** was prepared according to our previously reported method [27]. The crude product, which caused a strong mucous membrane irritation, could be used in the next step without further purification. To a solution of **B** (5.750 g, 27.00 mmol) in acetonitrile (100 mL), phthalimide potassium (5.000 g, 27.00 mmol) was added. The mixture was refluxing for 2 h, then concentrated under reduced pressure to afford compound **C** (4.878 g, 64%), white solid. m.p.:155.3-156.8, HRMS (ESI) m/z: 282.12381 [M + H]^+^, calcd. for C_16_H_15_N_3_O_2_ 282.12425. The compound **C** was purified by flash column chromatography and recrystallization from acetone [28]. The important intermediate **L** was obtained by the reaction of **C** (4.000 g, 14.23 mmol) and 80% hydrazine hydrate (N_2_H_4_ · H_2_O, 0.85 mL) in absolute ethyl alcohol (100 mL) refluxing for 5 h. The solution was filtered, concentrated under reduced pressure. The residue was dissolved in dichloromethane, filtered and recovered methylene chloride to give a pale yellow semi-solid substance **L** (3.521 g, 88%), HRMS (ESI) m/z: 152.11829 [M + H]^+^, calcd. for C_8_H_13_N_3_ 152.12827.

### Preparation of L1–L10

To a solution of **L** (1.324 mmol) and the corresponding cinnamic acids (1.322 mmol) in anhydrous CH_2_Cl_2_ (20 mL), EDCI (253 mg, 1.324 mmol) and triethylamine ((CH_3_CH_2_)_3_ N, 3.97 mmol) were added. The mixture was stirred at room temperature for 12 h. Then washed with water (2 × 20 mL) and brine (20 mL), successively, dried over sodium sulfate, filtered, and concentrated under vacuum. The residue was purified by flash chromatography and recrystallization from acetone.

*N-((3,5,6-trimethylpyrazin-2-yl)methyl)cinnamamide***(L1):** White solid, yield: 90.7%. HRMS (ESI) m/z: 282.15988 [M + H]^+^, calcd. for C_17_H_19_N_3_O 282.16064.

*(E)-3-(p-tolyl)-N-((3,5,6-trimethylpyrazin-2-yl)methyl)acrylamide***(L2):** White solid, yield: 83.5%. HRMS (ESI) m/z: 296.17554 [M + H]^+^, calcd. for C_18_H_21_N_3_O 296.17629.

*(E)-3-(3,4-dimethoxyphenyl)-N-((3,5,6-trimethylpyrazin-2-yl)methyl)acrylamide***(L3):** White solid, yield: 86.3%, m.p.: 150.8–151.4°C. ^1^H NMR (500 MHz, CDCl_3_): *δ* 7.62 (d, *J* = 15.6 Hz, 1H), 7.24 (s, 1H), 7.12 (d, *J* = 8.3 Hz, 1H), 7.07 (s, 1H), 6.86 (d, *J* = 8.3 Hz, 1H), 6.45 (d, *J* = 15.6 Hz, 1H), 4.61 (d, *J* = 3.7 Hz, 2H), 3.92 (s, 3H), 3.91 (s, 3H), 2.53 (s, 3H), 2.50 (br, 6H); ^13^C NMR (125 MHz, CDCl_3_): *δ* 166.2, 150.7, 149.8, 149.2, 147.9, 145.1, 141.0, 128.0, 122.1, 118.6, 111.2, 109.9, 56.1, 56.0, 41.4, 21.5, 21.5, 20.2. HRMS (ESI) m/z: 342.18167 [M + H]^+^, calcd. for C_19_H_23_N_3_O_3_ 342.18177.

*(E)-3-(4-methoxyphenyl)-N-((3,5,6-trimethylpyrazin-2-yl)methyl)acrylamide***(L4):** White solid, yield: 85.8%. HRMS (ESI) m/z: 312.17093 [M + H]^+^, calcd. for C_18_H_21_N_3_O_2_ 312.17120.

*(E)-3-(2-methoxyphenyl)-N-((3,5,6-trimethylpyrazin-2-yl)methyl)acrylamide***(L5):** White solid, yield: 90.1%, m.p.: 153.3–153.8°C. ^1^H NMR (500 MHz, CDCl_3_): *δ* 7.95 (d, *J* = 15.8 Hz, 1H), 7.52 (d, *J* = 7.5 Hz, 1H), 7.33 – 7.30 (m,1H), 7.25 (s, 1H), 6.96 – 6.93 (m, 1H), 6.91 (d, *J* = 8.3 Hz, 1H), 6.67 (d, *J* = 15.8 Hz, 1H), 4.61 (d, *J* = 3.9 Hz, 2H), 3.88 (s, 3H), 2.52 (s, 3H),2.51 (s, 3H), 2.50 (s, 3H); ^13^C NMR (125 MHz, CDCl_3_): *δ* 166.6, 158.3, 149.7, 148.0, 145.2, 136.5, 130.9, 128.9, 124.0, 121.5, 120.7, 111.2, 55.5, 41.4, 21.5, 20.2. HRMS (ESI) m/z: 312.17102 [M + H]^+^, calcd. for C_18_H_21_N_3_O_2_ 312.17120.

*(E)-3-(2,3-dimethoxyphenyl)-N-((3,5,6-trimethylpyrazin-2-yl)methyl)acrylamide***(L6):** White solid, yield: 83.6%, m.p.: 182.7–183.4°C. ^1^H NMR (500 MHz, CDCl_3_): *δ* 7.94 (d, *J* = 15.9 Hz, 1H), 7.31 (s, 1H), 7.16 (d, *J* = 7.8 Hz, 1H), 7.06 – 7.03 (m, 1H), 6.91 (d, *J* = 8.0 Hz, 1H), 6.64 (d, *J* = 15.9 Hz, 1H), 4.61 (d, *J* = 2.7 Hz, 2H), 3.87 (s, 3H), 3.85 (s, 3H), 2.51 (s, 3H), 2.50 (br, 6H); ^13^C NMR (125 MHz, CDCl_3_): *δ* 166.3, 153.3, 149.9, 148.4, 148.1, 148.0, 145.1, 135.8, 129.3, 124.3, 122.5, 119.5, 113.5, 61.4, 56.0, 41.5, 21.6, 21.6, 20.3. HRMS (ESI) m/z: 342.18155 [M + H]^+^, calcd. for C_19_H_23_N_3_O_3_ 342.18177.

*(E)-3-(3-methoxyphenyl)-N-((3,5,6-trimethylpyrazin-2-yl)methyl)acrylamide***(L7):** White solid, yield: 91.3%, m.p.: 117.2–117.9°C. ^1^H NMR (500 MHz, CDCl_3_): *δ* 7.64 (d, *J* = 15.6 Hz, 1H), 7.32 (s, 1H), 7.30 – 7.27 (m, 1H), 7.14 (d, *J* = 7.3 Hz, 1H), 7.06 (s, 1H), 6.90 (d, *J* = 7.9 Hz, 1H), 6.56 (d, *J* = 15.6 Hz, 1H), 4.60 (s, 2H), 3.83 (s, 3H), 2.53 (s, 3H), 2.50 (br, 6H); ^13^C NMR (125 MHz, CDCl_3_): *δ* 165.9, 159.9, 149.8, 148.0, 147.9, 144.9, 141.1, 136.3, 129.9, 121.0, 120.6, 115.5, 113.1, 55.4, 41.4, 21.5, 21.5, 20.2. HRMS (ESI) m/z: 312.17117 [M + H]^+^, calcd. for C_18_H_21_N_3_O_2_ 312.17120.

*(E)-3-(2,5-dimethoxyphenyl)-N-((3,5,6-trimethylpyrazin-2-yl)methyl)acrylamide***(L8):** White solid, yield: 88.5%. HRMS (ESI) m/z: 342.18118 [M + H]^+^, calcd. for C_19_H_23_N_3_O_3_ 342.18177.

*(E)-3-(3,4,5-trimethoxyphenyl)-N-((3,5,6-trimethylpyrazin-2-yl)methyl)acrylamide***(L9):** White solid, yield: 81.6%, m.p.: 154.7–155.4°C. ^1^H NMR (500 MHz, CDCl_3_): *δ* 7.60 (d, *J* = 15.5 Hz, 1H), 7.29 (s, 1H), 6.77 (s, 2H), 6.48 (d, *J* = 15.5 Hz, 1H), 4.61 (s, 2H), 3.89 (s, 6H), 3.87 (s, 3H), 2.53 (s, 3H), 2.50 (br, 6H); ^13^C NMR (125 MHz, CDCl_3_): *δ* 165.9, 153.5, 149.9, 148.0, 147.9, 144.9, 141.2, 139.5, 130.5, 120.0, 105.1, 61.1, 56.3, 41.4, 21.6, 21.5, 20.2. HRMS (ESI) m/z: 372.19232 [M + H]^+^, calcd. for C_20_H_25_N_3_O_4_ 372.19233.

*(E)-3-(2,3,4-trimethoxyphenyl)-N-((3,5,6-trimethylpyrazin-2-yl)methyl)acrylamide***(L10):** White solid, yield: 85.2%, m.p.: 139.8–140.5°C. ^1^H NMR (500 MHz, CDCl_3_): *δ* 7.81 (d, *J* = 15.8 Hz, 1H), 7.26 (d, *J* = 8.6 Hz, 1H),7.24 (s, 1H), 6.68 (d, *J* = 8.6 Hz, 1H), 6.59 (d, *J* = 15.8 Hz, 1H), 4.61 (d, *J* = 1.8 Hz, 2H), 3.91 (s, 3H), 3.88 (s, 3H), 3.88 (s, 3H), 2.52 (s, 3H), 2.51 (br, 6H); ^13^C NMR (125 MHz, CDCl_3_): *δ* 166.6, 155.0, 153.2, 149.8, 148.0, 147.9, 145.2, 142.5, 136.1, 123.4, 122.0, 120.1, 107.6, 61.4, 61.0, 56.1, 41.4, 21.6, 21.5, 20.2. HRMS (ESI) m/z: 372.17950 [M + H]^+^, calcd. for C_20_H_25_N_3_O_4_ 372.19233.

### Preparation of L11–L15, L17–L21

Intermediate **L** (1.324 mmol) and the corresponding phenolic acids (1.322 mmol) were dissolved in anhydrous DMF (**L11** CH_2_Cl_2_, **L21** DMI) (20 mL), EDCI (253 mg, 1.324 mmol) and HOBt (59 mg, 0.44 mmol) were added. The mixture was stirred at room temperature under nitrogen atmosphere for 12 h. The reaction mixture was washed with water (2 × 20 mL) and brine (20 mL), dried over sodium sulfate, filtered, and concentrated under vacuum. The residue was purified by colume chromatography and recrystallization from methanol to give **L11**–**L15** and **L17**–**L21**.

*2-(((3,5,6-trimethylpyrazin-2-yl)methyl)carbamoyl)phenyl acetate***(L11):** White solid, yield: 68.9%. HRMS (ESI) m/z: 336.13163 [M + Na]^+^, calcd. for C_17_H_19_N_3_O_3_ 336.13241.

*4-hydroxy-3-methoxy-N-((3,5,6-trimethylpyrazin-2-yl)methyl)benzamide***(L12):** White solid, yield: 81.8%, m.p.: 171.3–172.0°C. ^1^H NMR (500 MHz, CDCl_3_): *δ* 7.84 (s, 1H), 7.52 (d, *J* = 1.5 Hz, 1H), 7.35 (dd, *J* = 8.2, 1.5 Hz, 1H), 6.95 (d, *J* = 8.2 Hz, 1H), 6.40 (s, 1H), 3.93 (s, 3H), 2.52 (br, 6H), 2.51 (s, 3H); ^13^C NMR (125 MHz, CDCl_3_): *δ* 167.0, 149.9, 149.0, 148.0, 148.0, 146.8, 145.2, 126.6, 120.0, 114.2, 110.6, 56.1, 41.5, 21.6, 21.5, 20.2. HRMS (ESI) m/z: 302.15002 [M + H]^+^, calcd. for C_16_H_19_N_3_O_3_ 302.15047.

*4-hydroxy-N-((3,5,6-trimethylpyrazin-2-yl)methyl)benzamide***(L13):** White solid, yield: 76.9%. HRMS (ESI) m/z: 272.13925 [M + H]^+^, calcd. for C_15_H_17_N_3_O_2_ 272.13990.

*3-hydroxy-N-((3,5,6-trimethylpyrazin-2-yl)methyl)benzamide***(L14):** White solid, yield: 78.4%, m.p.: 210.4–211.3°C. ^1^H NMR (500 MHz, DMSO-*d*_6_): *δ* 9.64 (s, 1H), 8.76 (t, *J* = 5.0 Hz, 1H), 7.31 – 7.19 (m, 3H), 6.90 (dd, *J* = 7.8, 1.3 Hz, 1H), 4.51 (d, *J* = 5.2 Hz, 2H), 2.46 (s, 3H), 2.41 (br, 6H); ^13^C NMR (125 MHz, DMSO-*d*_6_): *δ* 166.2, 157.3, 149.1, 147.9, 147.8, 147.4, 135.7, 129.3, 118.1, 117.8, 114.3, 42.1, 21.1, 21.1, 20.3. HRMS (ESI) m/z: 272.13913 [M + H]^+^, calcd. for C_15_H_17_N_3_O_2_ 272.13990.

*2-hydroxy-N-((3,5,6-trimethylpyrazin-2-yl)methyl)benzamide***(L15):** White solid, yield: 84.3%. HRMS (ESI) m/z: 272.13943 [M + H]^+^, calcd. for C_15_H_17_N_3_O_2_ 272.13990.

*(E)-3-(4-hydroxyphenyl)-N-((3,5,6-trimethylpyrazin-2-yl)methyl)acrylamide***(L17):** Little yellow solid, yield: 71.3%. HRMS (ESI) m/z: 296.14035 [M-H]^−^, calcd. for C_17_H_19_N_3_O_2_ 296.13990.

*(E)-3-(3-hydroxyphenyl)-N-((3,5,6-trimethylpyrazin-2-yl)methyl)acrylamide***(L18):** Little yellow solid, yield: 74.8%, m.p.:196.3–197.0°C. ^1^H NMR (500 MHz, DMSO-*d*_6_): *δ* 9.60 (s, 1H), 8.52 (t, *J* = 5.0 Hz, 1H), 7.35 (d, *J* = 15.8 Hz, 1H), 7.20 (t, *J* = 7.8 Hz, 1H), 6.97 (d, *J* = 7.5 Hz, 1H), 6.93 (s, 1H), 6.77 (d, *J* = 8.0 Hz, 1H), 6.65 (d, *J* = 15.8 Hz, 1H), 4.47 (d, *J* = 5.2 Hz, 2H), 2.46 (s, 3H), 2.43 (s, 3H), 2.42 (s, 3H); ^13^C NMR (125 MHz, DMSO-*d*_6_): *δ* 164.8, 157.7, 149.4, 148.0, 147.8, 147.1, 139.2, 136.1, 129.9, 121.7, 118.8, 116.7, 113.7, 41.7, 21.1, 21.0, 20.2. HRMS (ESI) m/z: 296.14037 [M-H]^−^, calcd. for C_17_H_19_N_3_O_2_ 296.13990.

*(E)-3-(2-hydroxyphenyl)-N-((3,5,6-trimethylpyrazin-2-yl)methyl)acrylamide***(L19):** Little yellow solid, yield: 77.2%, m.p.: > 210°C. ^1^H NMR (500 MHz, DMSO-*d*_6_): *δ* 10.03 (s, 1H), 8.47 (t, *J* = 5.0 Hz, 1H), 7.66 (d, *J* = 15.9 Hz, 1H), 7.42 (d, *J* = 7.5 Hz, 1H), 7.17 (t, *J* = 7.6 Hz, 1H), 6.88 (d, *J* = 8.1 Hz, 1H), 6.81 (t, *J* = 7.4 Hz, 1H), 6.74 (d, *J* = 15.9 Hz, 1H), 4.46 (d, *J* = 5.2 Hz, 2H), 2.46 (s, 3H), 2.43 (s, 3H), 2.42 (s, 3H); ^13^C NMR (125 MHz, DMSO-*d*_6_): *δ* 165.4, 156.3, 149.4, 148.0, 147.8, 147.3, 134.8, 130.5, 128.1, 121.6, 121.3, 119.3, 116.1, 41.7, 21.1, 21.0, 20.3. HRMS (ESI) m/z: 296.14047 [M-H]^−^, calcd. for C_17_H_19_N_3_O_2_ 296.13990.

*(E)-3-(4-hydroxy-3-methoxyphenyl)-N-((3,5,6-trimethylpyrazin-2-yl)methyl)acrylamide***(L20):** Little yellow solid, yield: 80.1%. HRMS (ESI) m/z: 326.15090 [M-H]^−^, calcd. for C_18_H_21_N_3_O_3_ 326.15047.

*(E)-3-(3,4-dihydroxyphenyl)-N-((3,5,6-trimethylpyrazin-2-yl)methyl)acrylamide***(L21):** Little yellow solid, yield: 64.3%, m.p.: > 210°C. ^1^H NMR (500 MHz, DMSO-*d*_6_): *δ* 9.43 (s, 1H), 9.20 (s, 1H), 8.42 (t, *J* = 4.9 Hz, 1H), 7.26 (d, *J* = 15.7 Hz, 1H), 6.96 (s, 1H), 6.83 (d, *J* = 7.9 Hz, 1H), 6.75 (d, *J* = 8.1 Hz, 1H), 6.43 (d, *J* = 15.7 Hz, 1H), 4.45 (d, *J* = 4.9 Hz, 2H), 2.45 (s, 3H), 2.42 (s, 3H), 2.41 (s, 3H); ^13^C NMR (125 MHz, DMSO-*d*_6_): *δ* 165.3, 149.3, 147.9, 147.8, 147.3, 147.3, 145.5, 139.5, 126.3, 120.4, 118.2, 115.8, 113.9, 41.7, 21.0, 21.0, 20.2. HRMS (ESI) m/z: 336.13140 [M + Na]^+^, calcd. for C_17_H_19_N_3_O_3_ 336.13241.

### Preparation of L16

The desired compound **16b** was gained according to the method described by Kojima and Tranchimand with minor modifications [29,30]. EDCI (0.253 g, 1.324 mmol) and HOBt (0.059 g, 0.44 mmol) were added to a stirred solution of **16b** (0.442 g, 1.324 mmol) and the intermediate **L** (1.324 mmol) in anhydrous CH_2_Cl_2_ (20 mL). The mixture was stirred at room temperature for 12 h. The mixture was washed with water (2 × 20 mL) and brine (20 mL), dried over sodium sulfate, filtered, and concentrated under vacuum. Flash chromatography afforded **16c** (0.566 g, 91.6%), white solid, m.p.: 127-128°C, HRMS (ESI) m/z: 490.21033 [M + Na]^+^, calcd. for C_29_H_29_N_3_O_3_ 490.21066. To a solution of **16c** (0.48 g, 1.028 mmol) in methanol (20 mL), was added Pd/C 10% (0.01 g). Then the suspension was stirred under hydrogen atmosphere at room temperature for 12 h. The mixture was filtered, washed with methanol (2 × 20 mL), and methanol evaporated under vacuum to afford **L16**.

*3,4-dihydroxy-N-((3,5,6-trimethylpyrazin-2-yl)methyl)benzamide***(L16):** White solid, yield: 93.7%, m.p.: > 210°C. ^1^H NMR (500 MHz, DMSO-*d*_6_): *δ* 9.45 (s, 1H), 9.13 (s, 1H), 8.52 (t, *J* = 5.0 Hz, 1H), 7.29 (s, 1H), 7.22 (d, *J* = 8.2 Hz, 1H), 6.74 (d, *J* = 8.2 Hz, 1H), 4.48 (d, *J* = 5.0 Hz, 2H), 2.45 (s, 3H), 2.41 (s, 6H); ^13^C NMR (125 MHz, DMSO-*d*_6_): *δ* 165.9, 149.0, 148.4, 147.8, 147.5, 144.8, 125.5, 119.0, 115.1, 114.8, 42.1, 21.0, 21.0, 20.2. HRMS (ESI) m/z: 288.12699 [M + H]^+^, calcd. for C_15_H_17_N_3_O_3_ 288.13482.

### Bio-evaluation methods

#### Protective effect on damaged differentiated PC12 cells

The PC12 cell line (pheochromocytoma) was purchased from Institute of Materia Medica of Chinese Academy of Medical Science. Cells were growed in RPMI 1640 medium supplemented with 5% (v/v) fetal bovine serum (FBS), 10% (v/v) heat inactivated horse serum and 100 U/mL penicillin-streptomycin (Thermo Technologies, USA) and incubated at 37°C in a humidified atmosphere of 5% CO_2_. When cells achieved the desired density of > 80% confluency original medium was removed and cells were maintained with serum-free media for 14 h. The cells were resuspended in new growth media which consisted of RPMI 1640 supplemented with 10% heat inactivated fetal bovine serum. Cells were plated on 96-well dishes pre-coated with poly-L-lysine at 7 × 10^3^ cells/well, differentiated by treated with 50 ng/mL NGF for 48 h. After these, the differentiated PC12 cells were pretreated with various concentrations (60, 30, 15, 7.5, 3.75 μM) of ligustrazine derivatives for 36 h. Then the cells were induced by CoCl_2_ (final concentration, 200 mM) for 12 h. Control differentiated cells were injected with new growth media at equal amounts. After MTT solution (20 μL, 5 mg/mL) was added to each well, the plate was incubated for a further 4 h at 37°C. The supernatant was removed carefully without disturbing the attached cells and formazan crystals were solubilized by adding 100 μL DMSO into each well. After shaking for additional 15 min at 37°C, the plates were read for optical density at 490 nm (Thermo Multiskan GO, USA). CoCl_2_ was dissolved in RPMI 1640 medium. ligustrazine derivatives were dissolved in DMSO [21,31-33].

The proliferation rates of damaged PC12 cells were calculated in the following formula [OD_490_ (Compd) − OD_490_ (CoCl_2_)]/[OD_490_ (NGF) − OD_490_ (CoCl_2_)] × 100%; the EC_50_ values were using the equation below: −pEC_50_ = log C_max_ − log 2 × (ΣP − 0.75 + 0.25P_max_ + 0.25P_min_), Where C_max_ = maximum concentration, ΣP = sum of proliferation rates, P_max_ = maximum value of proliferation rate and P_min_ = minimum value of proliferation rate [18-21,34].
